# Accessing new polymorphs and solvates through solvothermal recrystallization

**DOI:** 10.1107/S2052252521012835

**Published:** 2021-12-16

**Authors:** David R. Allan

**Affiliations:** a Diamond Light Source Ltd, Diamond House, Chilton, Oxfordshire OX11 0DE, United Kingdom

**Keywords:** high-pressure structural chemistry, solvothermal recrystallization

## Abstract

In high-pressure structural chemistry, recrystallization techniques have been shown to be a powerful way of generating new polymorphs and solvates. A recent study by Olejniczak *et al.* [ *IUCrJ* (2022), **9**, 49–54] on the pyridazine-based compound 6-chloro-1,2,3,4-tetrazolo[1,5-*b*]pyridazine (C_4_H_2_N_5_Cl or CTP) uses these solvothermal techniques to establish the phase behaviour of CTP and the effects of partial hydration.

In this issue of **IUCrJ** , Olejniczak *et al.* (2022 ▸) describe a study of the pyridazine-based compound 6-chloro-1,2,3,4-tetrazolo[1,5-*b*]pyridazine (C_4_H_2_N_5_Cl or CTP) to establish its structural and phase behaviour at pressure and temperature. The material is an example of a high-nitro­gen-content organic compound and these systems have a comparatively high density but, conversely, are not observed to have the expected relatively short intermolecular contacts within their crystal structures. In their study, Olejniczak *et al.* examine these apparently anomalous structural characteristics and establish, via high-pressure crystallization, the phase behaviour and the effects of partial hydration.

To gain a more complete understanding of the properties of a crystalline system it is crucial that the effects of perturbing it from its equilibrium state, under the influence of some external stimulus, is investigated. The stimulus could be delivered by a variety of different forms, such as photo excitation or an applied electric field, but it is the thermodynamic parameters of temperature and pressure that are arguably the most fundamental and provide the most powerful means of driving significant structural change. The application of temperature and pressure are the principal drivers for chemical reaction and molecular synthesis, which form the foundations of chemistry and the chemical sciences. In the Earth and planetary sciences, high-pressure and variable-temperature studies provide a means of uncovering phenomena that are otherwise not directly observable but are, nevertheless, of crucial importance for an understanding of geological processes at extreme depths. Indeed, with most directly observable mineralogy only possible at the surface, or penetrating to very shallow depths, it is research in the Earth and planetary sciences that has initiated and progressed many of the innovations in high-pressure crystal chemistry.

The development of apparatus to maintain materials at high hydro­static pressure has been an active area of research for many years with some of the earliest pioneering work conducted by Bridgman (Bridgman, 1941 ▸) using piston-cylinder devices with designs evolving to massive hydraulically driven Bridgman-anvil and, latterly, multi-anvil presses. These large devices remain at the heart of hydro­thermal and solvothermal synthesis in the Earth and materials sciences where samples are synthesized under high-pressure and high-temperature conditions to be subsequently quenched and recovered for further analysis – the ‘cook and look’ approach. Although there have been a range of developments to allow *in-situ* measurements from these hydraulic press devices, such as the Paris–Edinburgh cell, which can be used for diffraction studies using either X-rays or neutrons, it was not until the advent of the gasketed diamond-anvil cell during the late 1960s that *in situ* measurement of samples held at high-pressure became accessible to less specialist laboratories. In the intervening decades the diamond-anvil cell has become the standard tool for the generation of high pressures and has been applied to a range of experimental techniques, such as Brillouin scattering (Whitfield *et al.*, 1976 ▸), Raman scattering (Sharma, 1989 ▸), NMR measurements (Lee *et al.*, 1987 ▸) and, of course, X-ray diffraction with both powder and single-crystal methods (Weir *et al.*, 1969 ▸). Diamond-anvil cells lend themselves to high-temperature studies as they are amenable to direct heating via electrical resistive heating of either the cell body or the anvils (Zha & Bassett, 2003 ▸; Louvel *et al.*, 2020 ▸) and the transparency of the anvils allows extreme temperatures to be achieved via laser heating (Anzellini & Boccato, 2020 ▸). These methods allow the study of systems at similar conditions to those deep within the Earth’s mantle, and approaching those found within the core, while allowing sufficient access to the sample so that changes in crystal structure and phase relationships can be directly observed.

For high-pressure structural chemistry, such extremes of pressure and temperature are generally not required, as they can lead to molecular dissociation, and most studies are confined to fairly modest pressure and temperature regimes where the accurate variation in key bond lengths can be followed or structural phase relationships can be mapped. For studies using X-ray single-crystal diffraction methods, miniature Merrill–Bassett style diamond-anvil cells can be used (Merrill & Bassett, 1974 ▸) with the sample crystal contained and compressed within a suitable hydro­static medium. For systems that are liquids at ambient conditions, compression within the diamond-anvil cell will initiate crystallization (freezing) and single crystals can be grown by cycling the temperature of the cell close to the elevated melting point – in a process similar to that undertaken by Tauer & Lipscomb for methanol (Tauer & Lipscomb, 1952 ▸) and a range of similar low-melting-point molecular systems, where they grew crystals in capillaries by cooling to just below the onset of freezing. For these high-pressure studies, the body of the diamond-anvil cell can be heated by a device as simple as a hot-air gun and, once a suitable crystal has grown on cooling to room temperature, the cell transferred to the diffractometer. For systems that are crystalline solids at room temperature and where the onset of molecular dissociation occurs before the melt, new high-pressure polymorphs can be generated by first dissolving the material in a suitable solvent and by then using pressure to induce recrystallization. A single crystal can be grown from the aggregate by repeatedly cycling the temperature to selectively dissolve all but the largest of the crystallites. This process helps to overcome any kinetic barrier to the molecular rearrangement required for polymorph formation and borrows from the hydro­thermal techniques used with diamond-anvil cells to grow single crystals of hydrated minerals, such as the hydrate calcium carbonate mineral Ikaite (Lennie, 2005 ▸). In high-pressure structural chemistry, these recrystallization methods have been shown to be a powerful method for generating new polymorphs and solvates (Fabbiani *et al.*, 2005 ▸) and is of particular interest in a range of systems including pharmaceutical compounds (Fabbiani *et al.*, 2003 ▸).

The exemplary study of Olejniczak *et al.* reported in this issue comes in a remarkable and comprehensive span of work from this group who have been at the forefront of not only fundamental high-pressure structural chemistry but have also been providing a lead in technique development (Boldyreva, 2019 ▸). In this new work, they have used hydro­thermal recrystallization to examine the effects of hydration on the phase behaviour and molecular tautomerization of CTP. This study provides another highly valuable contribution to the field.
